# A review of COFRADIC techniques targeting protein N-terminal acetylation

**DOI:** 10.1186/1753-6561-3-S6-S6

**Published:** 2009-08-04

**Authors:** Petra Van Damme, Jozef Van Damme, Hans Demol, An Staes, Joël Vandekerckhove, Kris Gevaert

**Affiliations:** 1 Department of Medical Protein Research, VIB, B-9000 Ghent, Belgium; 2 Department of Biochemistry, Ghent University, B-9000 Ghent, Belgium

## Abstract

Acetylation of nascent protein N^α^-termini is a common modification among archae and eukaryotes and can influence the structure and function of target proteins. This modification has been studied on an individual protein or (synthetic) peptide level or on a proteome scale using two-dimensional polyacrylamide gel electrophoresis. We recently developed mass spectrometry driven proteome analytical approaches specifically targeting the amino (N) terminus of proteins based on the concept of diagonal reverse-phase chromatography. We here review how this so-called combined fractional diagonal chromatography (COFRADIC) technique can be used in combination with differential mass-tagging strategies as to both qualitatively and quantitatively assess protein N^α^-acetylation in whole proteomes.

## Background

Earlier proteome analyses aiming at characterizing the protein N-acetylation status primarily used two-dimensional protein gels to compare maps of acetylated and non-acetylated proteins. For instance, studies comparing the proteomes of wild-type yeast with mutant strains deficient for a specific N-acetyltransferase (NAT) complex revealed charge shifts due to the absence or the presence of positively charged α-amino groups [1]. Data generated from such studies added a lot to our understanding of the biology of protein N^α^-terminal acetylation however, it was also realized that intrinsic drawbacks of 2D polyacrylamide gel electrophoresis techniques could result in sketchy proteome maps in which mainly well-soluble and highly abundant proteins were detected.

More comprehensive proteome coverage was achieved by so-called gel-free proteomics techniques (reviewed in [2]). Here, proteomes are digested into peptides which are generally more soluble than their precursor proteins and can be readily identified and quantified by modern mass spectrometric techniques. Interestingly, some gel-free techniques enrich protein N-terminal peptides independent of their *in vivo *modification status (blocked or free) and may thus eventually be used to assess protein N^α^-terminal acetylation states in a more complete manner. Examples of such targeted gel-free techniques are briefly discussed in the following. Strong cation exchange (SCX) chromatography at low pH has been used to enrich α-amino blocked N-terminal peptides from a protein trypsin digest [3] and was recently applied by the lab of Albert Heck on complete proteome digests [4,5]. Other methods target and thus deplete non-N-terminal peptides (internal peptides), generally holding a free α-amino group, by biotinylation [6] or using amino-reactive groups on solid supports (isocyanate resins) [7].

Our lab developed several gel-free proteomic techniques based on the principle of diagonal electrophoresis/chromatography [8]. Briefly, whole proteome digests are first separated by reverse-phase (RP) HPLC into distinct fractions. Each primary fraction or a combination thereof is then treated with an enzyme or a chemical compound modifying the structure of a selected class of peptides. This modification reaction is chosen such that peptides holding such modified structures are differently retained by chromatographic columns. Thus, when such modified primary fractions are separated a second time under identical chromatographic conditions as during the primary separation, they separate from non-modified peptides and are isolated for LC-MS/MS analysis. Our method is termed COFRADIC (combined fractional diagonal chromatography) and was initially developed to isolate peptides holding the rare amino acid methionine [9].

In 2003 we published a COFRADIC method to isolate N-terminal peptides from whole proteome digests [10]. The central modification step here is the reaction of free α-amino groups of internal peptides with 2,4,6-trinitrobenzenesulfonic acid (TNBS). As a result internal peptides now acquire a very hydrophobic trinitrophenyl group and segregate from the TNBS-non-reactive α-amino-blocked N-terminal peptides. We recently upgraded our N-terminal COFRADIC approach by introducing 1) SCX at low pH to pre-enrich for N-terminal peptides and 2) an enzymatic reaction to open up pyroglutamyl peptides for TNBS reaction. When applied to extremely complex peptide mixtures, this improved procedure sorts a subset of peptides typically consisting of more than 95% out of true N-terminal peptides [11].

Proteolysis results in the generation of new protein N-termini and thus not surprisingly, N-terminal COFRADIC was thus far mainly used to characterize protein processing in different cell death models (e.g. [12]). However, we recently showed that combined with amino-directed mass tags, our technology can readily be used to characterize the substrates of the human and yeast NatA complex by studying the acetylation status of isolated N-termini [13]. In this review we discuss the general principle of N-terminal COFRADIC and illustrate how stable isotope tagging, alternative amino-directed modifiers and a simple methionine oxidation step can be used to qualitatively and/or quantitatively characterize protein N^α^-terminal acetylation.

## Methods

Full technical details on the isolation of N-terminal peptides using the COFRADIC technology are documented elsewhere [11] and we here only describe additional chemistries that lead to quantification of the degree of protein acetylation, segregation of the *in vivo *blocked and free variants of protein N-terminal peptides and enrichment of methionine-containing N-terminal peptides.

### Synthesis of N-hydroxysuccinimide esters

N-hydroxysuccinimide (NHS) esters of D_3_-acetate (Sigma-Aldrich, St. Louis, MO, USA) and propionate (Sigma-Aldrich) were synthesized according to [14]. An NHS ester of ^13^C_2_D_3_-acetate was synthesized as follows. 1.06 g N-hydroxysuccinimide (Sigma-Aldrich) was dissolved in 10 ml ethyl acetate. Then, 2.68 g diphenylchlorophosphate (Sigma-Aldrich) in 10 ml ethyl acetate was added, followed by drop-wise addition of 2.11 g triethylamine (Sigma-Aldrich) in 10 ml ethyl acetate (temperature was at 40°C). A thick white suspension formed to which 0.5 g ^13^C_2_D_4 _acetic acid (Sigma-Aldrich) in 10 ml ethyl acetate was added and which was then heated to 50°C. After stirring for 16 hours, the solid precipitate was filtered and washed twice with 3 ml ethyl acetate. The combined ethyl acetate filtrates were dried *in vacuo*. The resulting residue was dissolved in 20 ml ethanol at 50°C. After cooling to -18°C for two hours, the precipitate was filtered and washed twice with 2 ml of isopropanol. After drying *in vacuo*, 1.1 g of NHS-^13^C_2_D_3_-acetate was obtained.

### Oxidation of methionine to methionine-sulfoxide

Methionines are uniformly converted to their methionine-sulfoxide derivatives by transferring 2 μl of a freshly prepared aqueous 30% (w/v) H_2_O_2 _solution to the peptide containing fraction(s) which are dissolved in 100 μl of 10 mM ammonium acetate and 5% acetic acid. The oxidation reaction is allowed to proceed for 30 min at 30°C, after which the sample is immediately injected onto the RP-HPLC column.

## Results and discussion

### The general concept of isolating N-terminal peptides by COFRADIC

**Figure 1:**
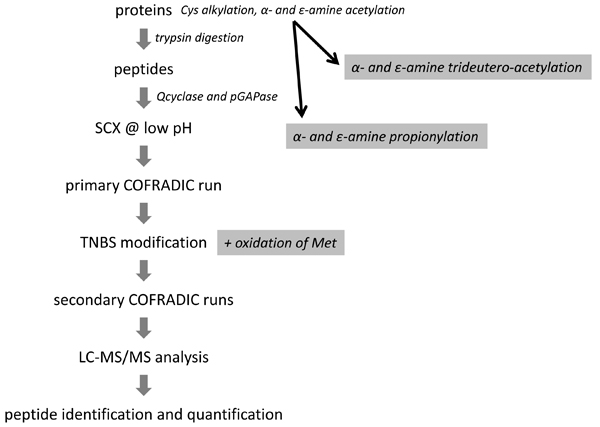
**General work-flow of the N-terminal COFRADIC procedure**.

We here combine the major steps of the workflow for the N-terminal COFRADIC procedure (Figure 1) and refer to [10,11] in which full experimental details are described.

Prior to trypsin digestion, proteins are denatured in high concentrations of chaotropes (e.g. 4 M guanidinium hydrochloride), protein disulfide bridges are reduced and free thiol groups are alkylated using iodoacetamide. Next, all free primary amines (α- and ε-amines) are blocked by acetylation. Thus, when trypsin is added, it is only able to cleave after arginines since acetylated lysines are not recognized. In other words, trypsin is now acting as endoproteinase Arg-C thus yielding peptides ending on arginine. Protein N-terminal peptides further differ from all other peptides since they carry an acetylated (either *in vivo *or acquired *in vitro*) α-amino group. In our original setup this chemical difference was exploited by targeting internal peptides (carrying a free α-amino group) by TNBS [10]. And, more recently, by further introducing an additional SCX step to enrich for blocked N-terminal peptides. Indeed, at pH 3, these peptides carry one positive charge less than internal peptides and thus interact significantly weaker with SCX resins [11].

It is important to note that the actual sorting reagent, TNBS, only reacts with primary amines and not with peptides starting with proline or pyroglutamyl residues. Combining glutamine cyclotransferase and pyroglutamyl aminopeptidase significantly reduces the background of pyroglutamyl peptides. The former drives formation of pyroglutamate to completion, whereas the latter efficiently cleaves it from the peptide except when proline is the second amino acid [15]. Obviously, removal of pyroglutamyl residues can be done prior to or following the SCX enrichment step however, more conveniently it is done before SCX upon which the shortened peptides are retained by the SCX resin.

The actual COFRADIC step consists of a primary RP-HPLC fractionation of the SCX enriched peptide fraction. Each primary fraction is then reacted with TNBS and internal peptides obtain a trinitrophenyl group, making them more hydrophobic. During a series of secondary, identical RP-HPLC separations of such TNBS-modified fractions, the modified internal peptides segregate out of the primary collection intervals whereas the unmodified N-terminal peptides elute within the same time-frame and are collected for LC-MS/MS analysis.

To characterize N^α^-acetylation, and as such NAT substrates in detail, we introduced new chemistries in the N-terminal sorting procedure enabling quantitative analysis of protein N^α^-terminal acetylation or more targeted isolation of NatB and NatC substrates. These chemistries are discussed in the following sections.

### Trideutero-acetylation of free protein N^α^-termini: analysis of the degree of protein N^α^-terminal acetylation

**Figure 2:**
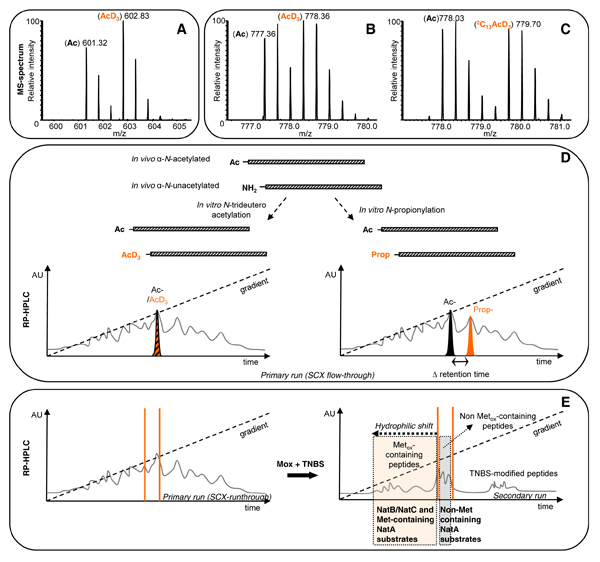
**A, B and C: Representative MS spectra of N-terminal peptides from partially alpha-N-acetylated proteins**. The MS-spectrum from the N-terminal peptide (doubly charged precursor) of the DnaJ homolog subfamily C member 2 (^1^MLLLPSAADGR^11^, 44% acetylated) reveals two distinguishable isotopic envelopes of the two isotopomers (i.e. the acetylated (Ac) and trideutero-acetylated forms (AcD_3_)) (A). Panels B & C show MS-spectra of the N-terminal peptide (triply charged precursor) of the signal recognition particle 68 kDa protein (^2^AAEKQVPGGGGGGGSGGGGGSGGGGSGGGR^31^) when trideutero-acetylated (B) or ^13^C_2_D_3_-acetylated (C) (53% acetylated). Panel B illustrates the need for increased mass spacing by heavier isotopomers. **D: Comparison of protein N-trideuteroacetylation and N-propionylation**. A protein that is partially *in vivo *α-*N*-acetylated can be modified *in vitro *by *N*-trideutero-acetylation or *N*-propionylation. When trideutero-acetylation is used, the RP-HPLC elution profiles of the α-*N*-acetylated and α-*N*-trideutero-acetylated variants are indistinguishable and the peptide variants only segregate upon MS analysis by their 3 Da mass difference. In contrast, when *N*-propionylation is used the α-*N*-acetylated and α-*N*-propionylated variants segregate upon RP-HPLC, with the propionylated variant eluting at a later time because of increased hydrophobicity. **E.: The use of methionine oxidation to segregate methionine-containing from non-methionine-containing N-terminal peptides**. Methionine oxidation, when applied in between the primary and secondary RP-HPLC separations and after TNBS modification, causes methionine-sulfoxide containing N-terminal peptides to shift to earlier elution times on RP-HPLC. As a result the N-terminal peptides of NatB (Met-Asn, Met-Asp and Met-Glu) and NatC (Met-Ile, Met-Leu and Met-Phe) substrates, as well as of substrates of yet unidentified NATs (e.g. Met-Lys) migrate out of the primary collection intervals and are in this way enriched from the non-methionine-containing NatA substrates.

The group of Fred Regnier previously reported on the use of trideutero-acetylation of so-called signature peptide N-termini for quantitative proteomics [14]. However, when this reaction is performed at the protein level rather than the peptide level (Figure 1), one can distinguish between *in vivo *acetylated and their chemically trideutero-acetylated N-terminal counterparts (*in vivo *free protein N-termini) [11]. Both types of peptides behave extremely similarly during all enrichments steps and only segregate during mass spectrometric analysis. Thus, in MS-spectra very often these two types of N-terminal peptides are easily distinguished (Figure 2A) and the relative amount of each of these can be weighed, which finally provides information on the degree of protein N^α^-terminal acetylation [13].

Different groups previously pointed to small hydrophobicity differences existing between deuterated and naturally hydrogenated isotopomers, causing the deuterated ones to elute somewhat sooner from reverse-phase columns compared to their hydrogenated counterparts (e.g. [16]). However, we found only minor, if any differences in the elution patterns of acetylated or trideutero-acetylated N-terminal peptides, thus complying with further quantification. In general, a difference in the N^α^-acetylation level of about 5% can be accurately quantified. Nevertheless, since mass spacing between acetylated and trideutero-acetylated N-terminal peptides is only 3 atomic mass units for lysine-free N-terminal peptides, overlapping isotopic envelopes are expected for peptides of more than 1000 Da, and generally this makes quantification of the degree of *in vivo *acetylation for peptides of about 3000 Da (i.e. peptides of which the intensity of the fourth isotopic peak from the theoretical isotopic envelope, equals or supersedes the intensity of the mono-isotopic peak) difficult and even impossible when using low-resolution mass spectrometry (see Figure 2B). This caveat however, can be overcome by using an N-hydroxysuccinimide ester of deuterated and carbon-13 acetic acid, which will evoke a mass difference of 5 atomic mass units per primary amine, which is generally sufficiently large to allow quantification (Figure 2C).

### Segregating blocked and free protein N^α^-termini peptides by propionylation

An alternative to ^13^C_2_D_3_-acetylation is propionylation of primary amino groups. Propionylated peptides are however somewhat more hydrophobic than their acetylated counterparts and thus segregate on RP-HPLC columns from their acetylated forms (Figure 2D), as such leading to less complex MS-spectra of partially *in vivo *α-N-acetylated N-termini. Hence, in this way *in vivo *free and blocked protein N-termini can be identified and substrates of NATs can be qualitatively characterized. However, within one analyzed N-terminal proteome, the actual determination of the degree of acetylation is not straightforward. First of all, one may well expect (small) differences in the net efficiency of ionization of acetylated or propionylated peptides, implying that the intensities of the isotopic envelopes of both types of peptides may not be directly compared. Secondly, because of the imposed segregation of both types of peptides as well as the high complexity of the analyte mixture, acetylated and propionylated peptides co-elute with different "neighbouring" peptides that affect each others' ionization efficiency (i.e. by ionization suppression effects), again not allowing direct weighing of the degree of protein N^α^-terminal acetylation.

Inter-experimentally (e.g. when comparing two proteomes in one experiment), propionylation of free protein N^α^-termini could however be used to determine changes of the degree of protein acetylation; for example when comparing metabolically arginine-labeled peptides [17] from two different proteomes (e.g., control versus perturbation of a particular NAT activity). In this case, arginine-labeling ensures that isolated N-terminal peptides can be quantified [18]. If no further changes in total protein concentration are expected (e.g. by blocking protein synthesis and/or degradation), isotopic envelopes of *in vivo *acetylated and/or *in vitro *propionylated (*in vivo *free) peptides may be compared and point to possible fluxes of protein acetylation.

### Enrichment of Met-containing N-terminal peptides

Methionine is very susceptible to oxidation: its sulfur atom readily oxidizes to a sulfoxide derivative when exposed to air. We previously suggested oxidizing all methionyl peptides to methionine-sulfoxide peptides before starting the primary COFRADIC separation thereby creating uniform and oxidation-stable peptide mixtures [11]. However, this oxidation step can also be introduced between the primary and secondary RP-HPLC separation, thereby causing all methionine-containing N-terminal peptides to shift to earlier elution times (Figure 2E). As a result, the N-terminal peptides of NatB (Met-Asn, Met-Asp and Met-Glu) and NatC (Met-Ile, Met-Leu and Met-Phe) substrates, as well as of substrates of yet unidentified NATs (e.g. Met-Lys) migrate out of the primary collection intervals and are in this way enriched. Clearly, N-terminal peptides containing an internal methionine will also elute earlier, but such N-termini typically only represent 20% of the total of NatA-type substrate N-termini identified. Moreover, the increased separation of peptides typically leads to increased overall proteome coverage (e.g. [19]).

## Conclusion

We here reviewed different ways by which the N-terminal COFRADIC technique can be fine-tuned to study protein N^α^-terminal acetylation. Protein trideutero-acetylation is used to mass tag all primary protein amino groups including *in vivo *free N^α^-termini and can thus be used to quantify the degree of *in vivo *protein acetylation by interpreting the MS spectra of isolated protein N-terminal peptides. If the isotopic envelopes of these isotopomers overlap too heavily, amino modification using a deuterated and carbon-13 variant (^13^C_2_D_3_-acetic acid) will increase the mass difference between the two N-terminal peptide variants to 5 atomic mass units (instead of 3) and can thus be used to study the degree of protein acetylation in a proteome. When protein acetylation between different proteomes needs to be compared, we suggest propionylation instead of acetylation. Propionylated N-terminal peptides (originating from *in vivo *free protein forms) segregate from acetylated counterparts and allow inter-proteome analysis of the degree of protein acetylation. Finally, the substrates of NatB and NatC acetyltransferases may be more specifically targeted by a methionine oxidation step prior to the secondary COFRADIC runs: these peptides start with an acetylated methionine residue that is rendered more hydrophilic by oxidation (to the sulfoxide form) and thereby segregate from the majority of NatA substrates.

In conclusion, we here illustrated the flexibility and versatility of N-terminal COFRADIC for targeted qualitative and/or quantitative characterization of N^α^-terminal protein acetylation and believe that this technology should be placed at the forefront of protein acetylation research.

## Competing interests

The authors declare that they have no competing interests.

## Authors' contributions

PVD, JV and KG designed the proteome studies and PVD performed the COFRADIC analyses and data interpretation. JVD was in charge of LC-MS/MS analysis of isolated peptides. AS and HD synthesized the different amino-reactive reagents. PVD, JV and KG wrote the manuscripts and all authors read and corrected it.
